# Immune microenvironment composition in non‐small cell lung cancer and its association with survival

**DOI:** 10.1002/cti2.1142

**Published:** 2020-06-12

**Authors:** Menno Tamminga, Thijo Jeroen N Hiltermann, Ed Schuuring, Wim Timens, Rudolf SN Fehrmann, Harry JM Groen

**Affiliations:** ^1^ Department of Pulmonary Diseases University Medical Center Groningen University of Groningen Groningen The Netherlands; ^2^ Department of Pathology and Medical Biology University Medical Center Groningen University of Groningen Groningen The Netherlands; ^3^ Department of Medical Oncology University Medical Center Groningen University of Groningen Groningen The Netherlands

**Keywords:** adenocarcinoma, biostatistics, immune microenvironment, non‐small cell lung cancer, smoking, squamous cell carcinoma

## Abstract

**Objectives:**

In non‐small cell lung cancer (NSCLC), the immune system and possibly its composition affect survival. In this *in silico* study, the immune infiltrate composition in NSCLC patients was evaluated.

**Methods:**

Gene expression data of tumors from early NSCLC patients were obtained from Gene Expression Omnibus (GEO). With CIBERSORT, 22 immune cell fractions were estimated.

**Results:**

The immune infiltrate of 1430 pretreatment NSCLC patients contained mostly plasma cells, macrophages and CD8 T cells. Higher fractions of resting mast and CD4 T‐helper cells were associated with longer overall survival (OS) (HR = 0.95, *P* < 0.01; HR = 0.98, = 0.04, respectively) and higher fractions of M2 macrophages and active dendritic cells with shorter survival (HR = 1.02, *P = *0.03; HR = 1.03, *P = *0.05, respectively). Adenocarcinoma patients with survival data (*n* = 587) showed higher fractions of resting mast and resting CD4 T cells, and lower M0 macrophages than squamous cell carcinoma (*n* = 254), which were associated with OS (HR = 0.95, *P = *0.04; HR = 0.97, *P = *0.01; HR = 1.03, *P = *0.01, respectively). Fractions of memory B cells, naïve CD4 T cells and neutrophils had different associations with survival depending on the subtype. Smokers had had higher fractions of regulatory T cell, follicular helper T cell, neutrophil and M2 macrophage, which were associated with shorter survival (HR = 1.3, *P* < 0.01; HR = 1.13, *P = *0.02; HR = 1.09, *P = *0.03; HR = 1.04, *P = *0.02, respectively).

**Conclusion:**

Pretreatment differences in immune cell composition in NSCLC are associated with survival and depend on smoking status and histological subtype. Smokers' immune composition is associated with lower survival.

## Graphical abstract

Because of small populations, it is difficult to assess differences in the immunocomposition and their associated survival depending on smoking behaviour and tumor subtype for non‐small cell lung cancer patients. Here, we used an *in silico* approach of all available material to identify these differences and the associations with survival. Immune cell fractions showed different, sometimes opposing associations depending on the tumor subtype and smoking status.

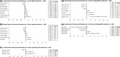

## Introduction

In non‐small cell lung cancer (NSCLC) patients, the immune system plays an important role in both the response to therapy and overall survival.^1^, ^2^, ^3^, ^4^, ^5^ The upregulation of programmed death‐ligand 1 (PD‐L1) on tumor cells in biopsies and the interaction of these tumor cells with T cells are associated with tumor response to immune‐modulating therapies. Unfortunately, tumor‐response percentages only reach 20–40% even in the best studies.^3^, ^4^, ^6^, ^7^, ^8^, ^9^

Increased numbers of tumor infiltrating lymphocytes (TILS), especially cytotoxic CD8 T cells and CD4 helper T cells, have been associated with responding tumors and improved survival, while higher numbers of regulatory T cells protect tumors against the native immune system.^10^, ^11^, ^12^, ^13^ Other levels of complexity come from subtypes of lymphocytes which have a different effect on survival. Other cell types, such as tumor‐associated macrophages and tumor‐associated neutrophils (TAMs and TANs) and their subtypes, have their own prognostic effects.^12^, ^14^, ^15^, ^16^, ^17^, ^18^

Although larger studies differentiate between histological subtypes, many small studies that investigate the effect of immune cells often pool all NSCLC patients in one group. NSCLC is predominantly characterised by two different histological subtypes: adenocarcinoma and squamous cell carcinoma. It is known that each subtype has different driver mutations and different immune genes that are activated.^19^, ^20^, ^21^ Although the tumor response on immune‐modulating therapy is similar in both subtypes, the underlying immune mechanism may be different. This may be reflected by different immune cell compositions. Another important prognostic factor is smoking status. Smoking is the major environmental event that causes lung cancer. Survival is decreased in smokers; however, chances to respond to immune‐modulating therapy are increased. A possible explanation is that tumors of smokers have an increased mutational burden which has been associated with stimulation of the immune system by neoantigens.^4^, ^22^, ^23^

While specific individual immune cells have been well studied, the role of the immune composition is less well investigated.^9^, ^10^, ^11^, ^12^, ^13^, ^14^, ^15^, ^16^, ^17^, ^18^, ^24^ Due to the often small number of patients in many studies, differences between subtype and smokers/non‐smokers could not be evaluated. In this *in silico* study, we evaluated the immune microenvironment in mainly early pretreatment NSCLC patients. Differences in immune composition for subtype and smoking status, and implications for survival were studied.

## Results

### NSCLC patients

**Figure 1:**
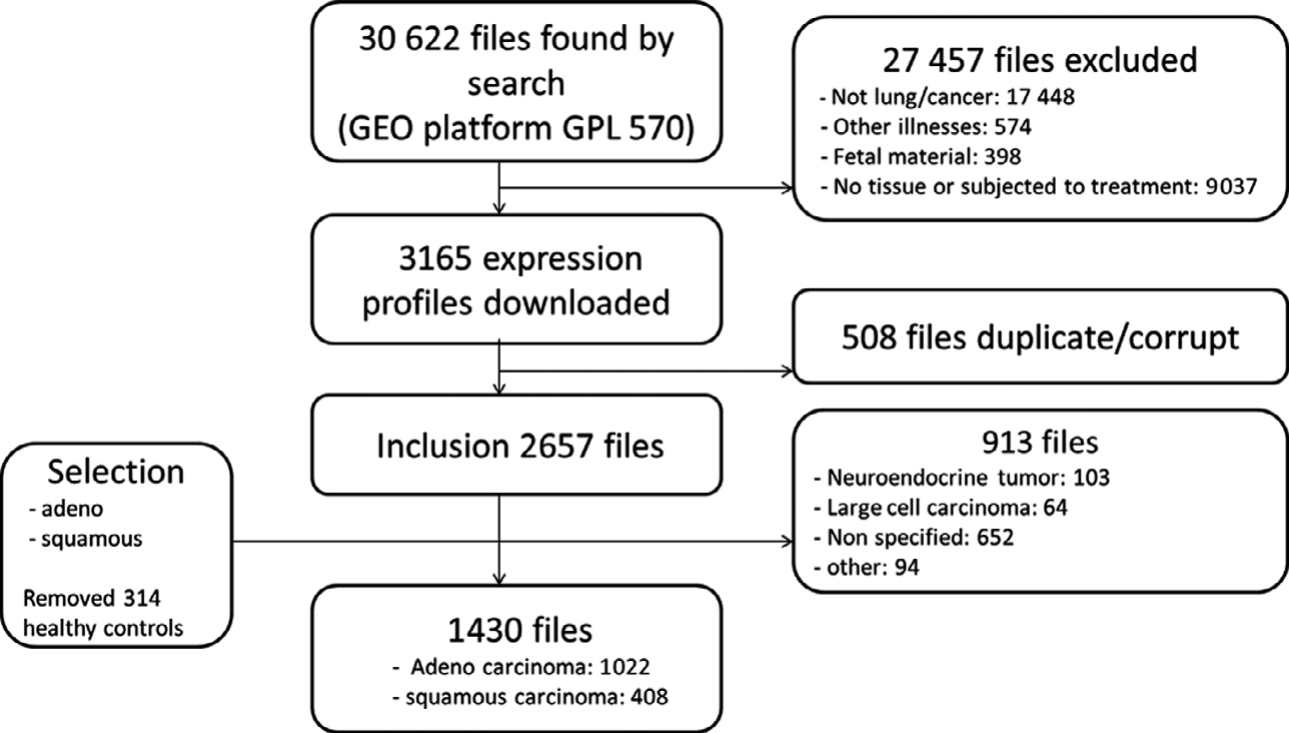
Flowchart of sample acquisition.

We evaluated 1742 samples from 22 different studies (Supplementary table 3). Among these, 1430 samples from NSCLC patients were identified (Figure 1). Adenocarcinoma made up the majority of patients (*n* = 1022, 71.5%). Patients had a median age of 64 (range 30–93) years and were mostly male (62%) and smokers (76%) (Supplementary table 4). The majority of patients had early‐stage disease (*n* = 922, 654 had stage I, and 268 had stage II), with only 62 having advanced stages (stage III = 54 and stage IV = 8). For the remaining 446 patients, no stage data were available. OS data were available for 841 patients. These patients had a median survival of 7.2 years (95% confidence interval: 6.3–8.1 years) (Supplementary figure 1). They primarily had early‐stage disease (stage I = 398, stage II = 143), with a minority having advanced stages (26 stage Ill, and 4 stage IV). Stage of disease was missing for 270 patients with survival data. Patients with and without OS data did not differ in their characteristics from the whole population.

### Distribution of immune cell infiltrate in NSCLC tumors

**Figure 2:**
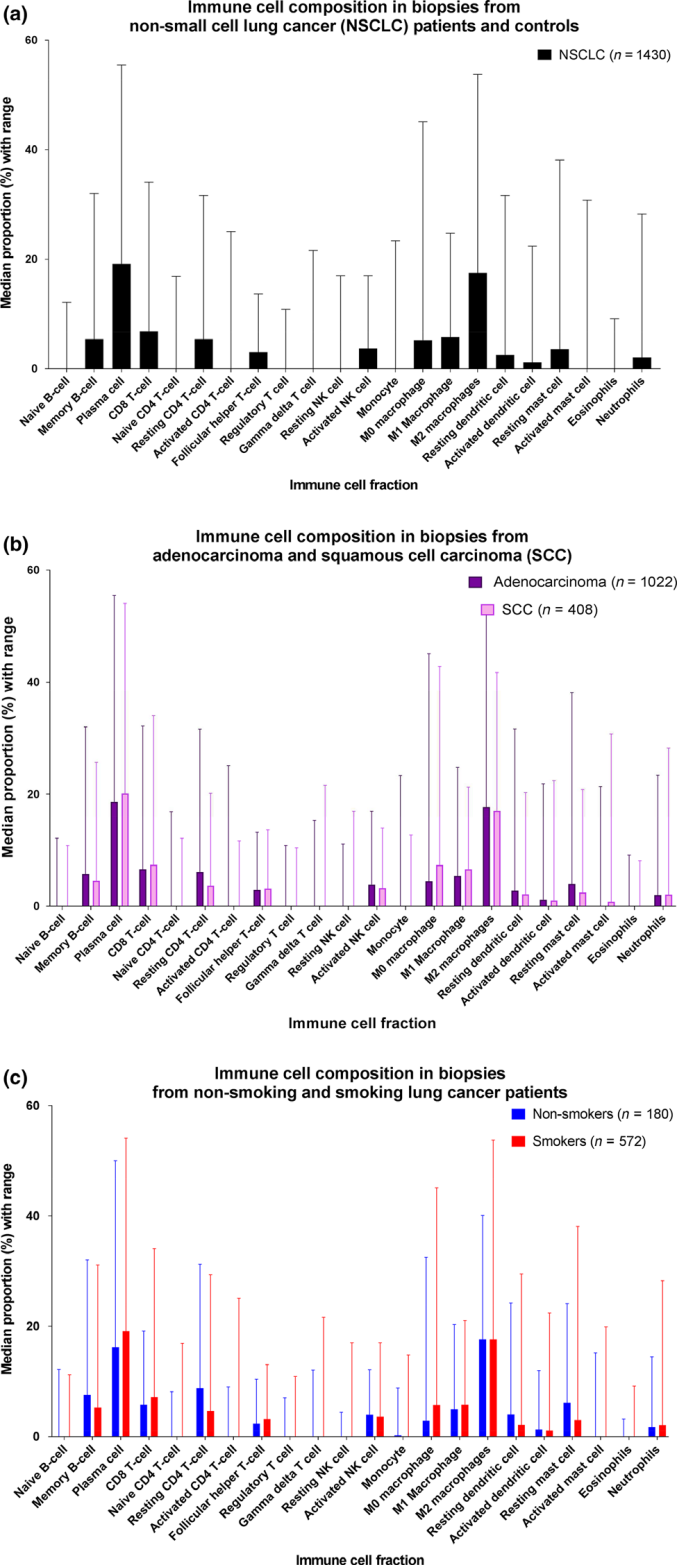
Immune infiltrate composition for **(a)** non‐small cell lung cancer patients, **(b)** adenocarcinoma and squamous cell carcinoma, and **(c)** smokers and non‐smokers. Large interpatient variations were observed. Between adenocarcinoma (dark purple) and squamous cell carcinoma (light purple), all immune cell fractions except for the eosinophil, neutrophil, active dendritic cell and resting CD4 T‐cell fraction were significantly different. While between smokers (red) and non‐smokers (blue), no significant differences were found for fractions of naive CD4 T cells, gamma delta T cells, NK cells (resting and active), M1 and M2 macrophages, active dendritic cells and eosinophils. Patient numbers are for those with histiotype or known smoking history.

The majority of the immune infiltrate in tumors of NSCLC patients was made up of plasma cells, and M2 macrophages (Figure 2, Supplementary table 5), followed by M0/M1 macrophages, CD8 T cells, resting CD4 T cells, mast cells and memory B cells. The immune composition showed large interpatient variations, both in individual immune cell fractions and in the whole immune composition (Figure 2, Supplementary table 5).

### Immune cell fractions in NSCLC tumors and overall survival

**Figure 3:**
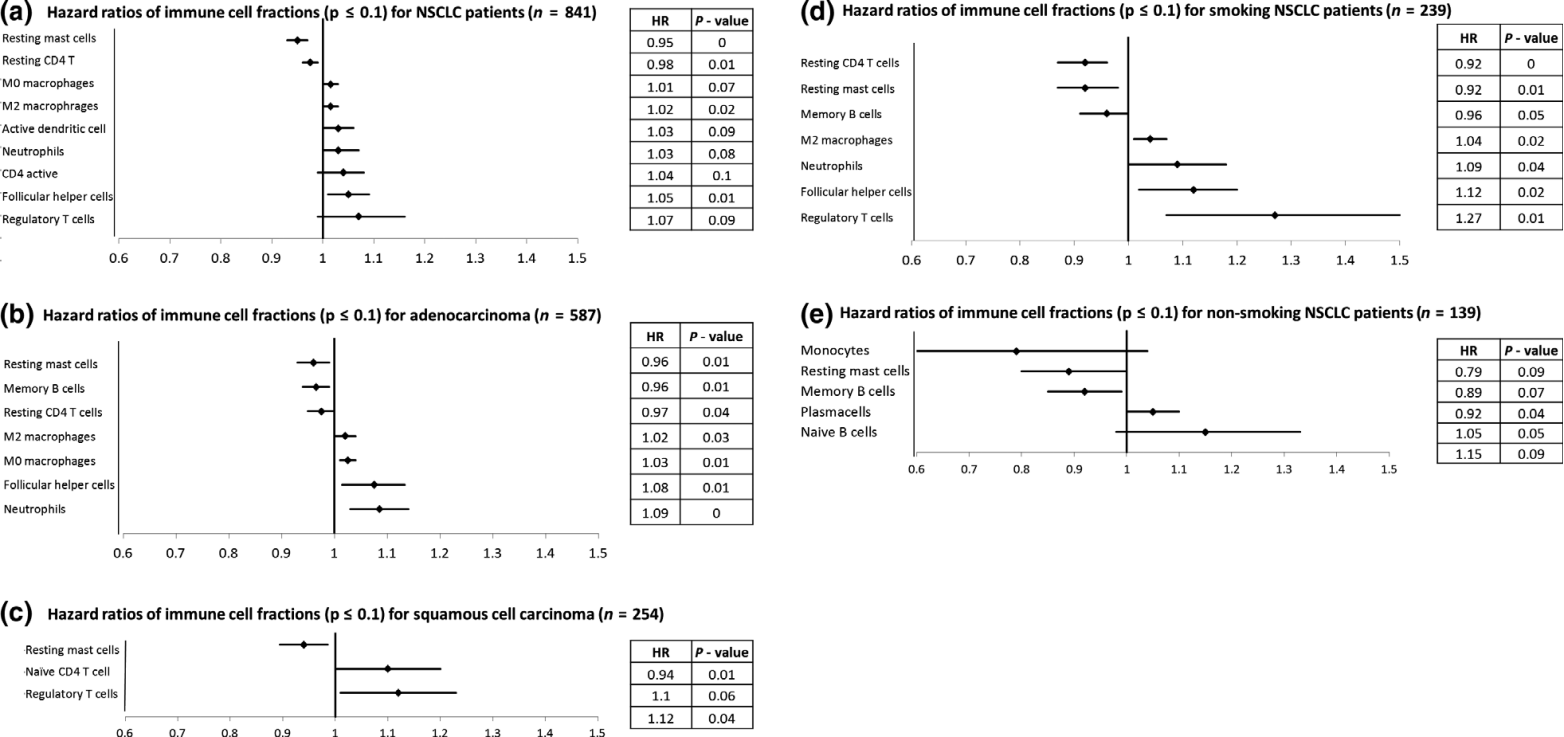
Association of different immune cell fractions with overall survival **(a)**, stratified for histological subtype **(b, c)** and smoking status (current and past smoking) **(d)** versus never smoking **(e)**. HR > 1 indicates a higher proportion of immune cells being associated with worse survival, while a HR < 1 is associated with better outcome. The HR indicates the risk associated with an increase of 1 percentage point of the immune cell fraction, and stacks for further changes.

**Figure 4:**
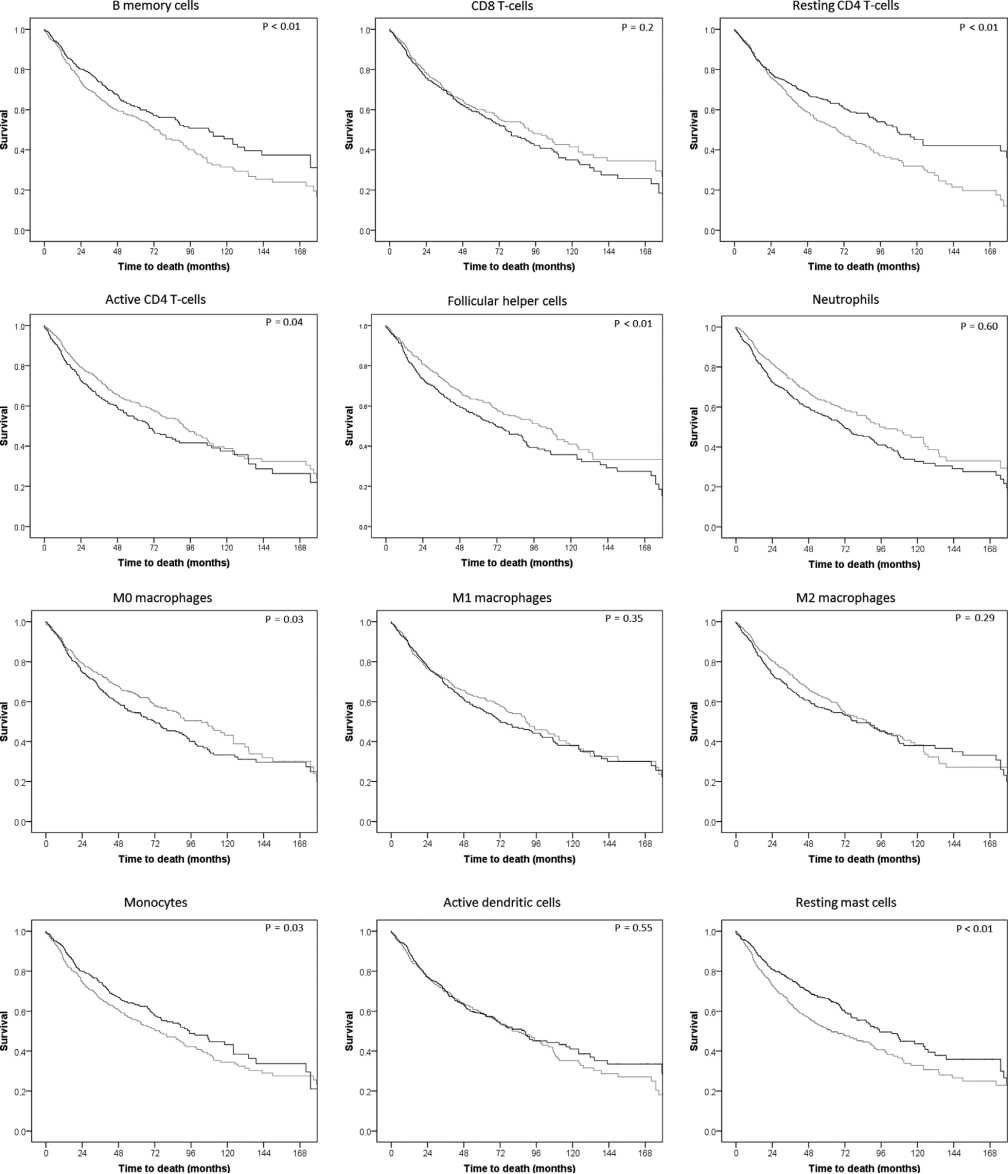
Survival curves of NSCLC (*n* = 841) patients by high and low expression of different immune cell fractions. Immune cell fractions are those with survival outcomes, with the exception of the regulatory T‐cell and active CD4 T‐cell fractions as these could not be split by their median into two equal groups. Black depicts the patient group with a proportion of immune cells above the median, grey below the median. *P*‐values are those of the log‐rank test comparing the two groups split by the median proportion of the immune cell fraction.

A higher fraction of resting mast cells and resting CD4^+^ T cells was significantly associated with a longer overall survival (HR = 0.95, *P* < 0.01; HR = 0.98, *P = *0.01, respectively), while a higher fraction of follicular helper cells and M0 macrophages was associated with shorter overall survival (HR = 1.05, *P = *0.01; HR = 1.02, *P = *0.02, respectively) (Figures 3, 4, Supplementary table 6). The plasma cell fraction was not associated with survival (HR = 0.99, *P = *0.17).

When testing for an interaction of immune cell fractions between adenocarcinoma and squamous cell carcinoma, significant interactions were observed for memory B‐cell fraction (HR = 0.96, for every percentage point increase in memory B‐cell fraction, the HR of all NSCLC patients decreases by 0.04), interaction (HR = 1.07, for every percentage point increase in memory B‐cell fraction, the HR of squamous cell carcinoma patients increases by 0.07, *P* < 0.01), neutrophil fraction (HR = 1.09, interaction HR = 0.91, *P = *0.01), and naïve CD4 T‐cell fraction (HR = 0.92, interaction HR = 1.21, *P = *0.02). This shows that higher B‐cell and naïve CD4 T‐cell fractions were associated with better OS in adenocarcinoma, while higher fractions in squamous cell carcinoma are associated with worse OS. The neutrophil fraction was not associated with survival in squamous cell carcinoma in any way, while in adenocarcinoma they were an unfavorable sign. For smoking status, only a stratified analysis was performed, as there were too few patients in the non‐smoking group to accurately investigate any interactions.

### Immune cell infiltrate by histological subtype

Twelve cell fractions differed significantly (*P* < 0.0022) between adenocarcinoma and squamous cell carcinoma. In adenocarcinoma (compared to squamous cell carcinoma), the largest positive difference in percentage points was observed in the resting CD4 T‐cell (+2.4 percentage point), resting mast cell (+1.5 percentage point), memory B‐cell (+1.1 percentage point) and active NK cell (+0.6 percentage point) fractions. The largest negative difference was observed for the M0 macrophage (−2.8 percentage point), plasma cell (−1.5 percentage point), M1 macrophage (−1.2 percentage point), active mast cells (−0.8 percentage point) and resting dendritic cell (−0.6 percentage point) fractions. Fractions of naïve B cells, resting NK cells and monocytes differed significantly in their distribution.

### Survival impact of immune cell fractions by histological subtype

**Figure 5:**
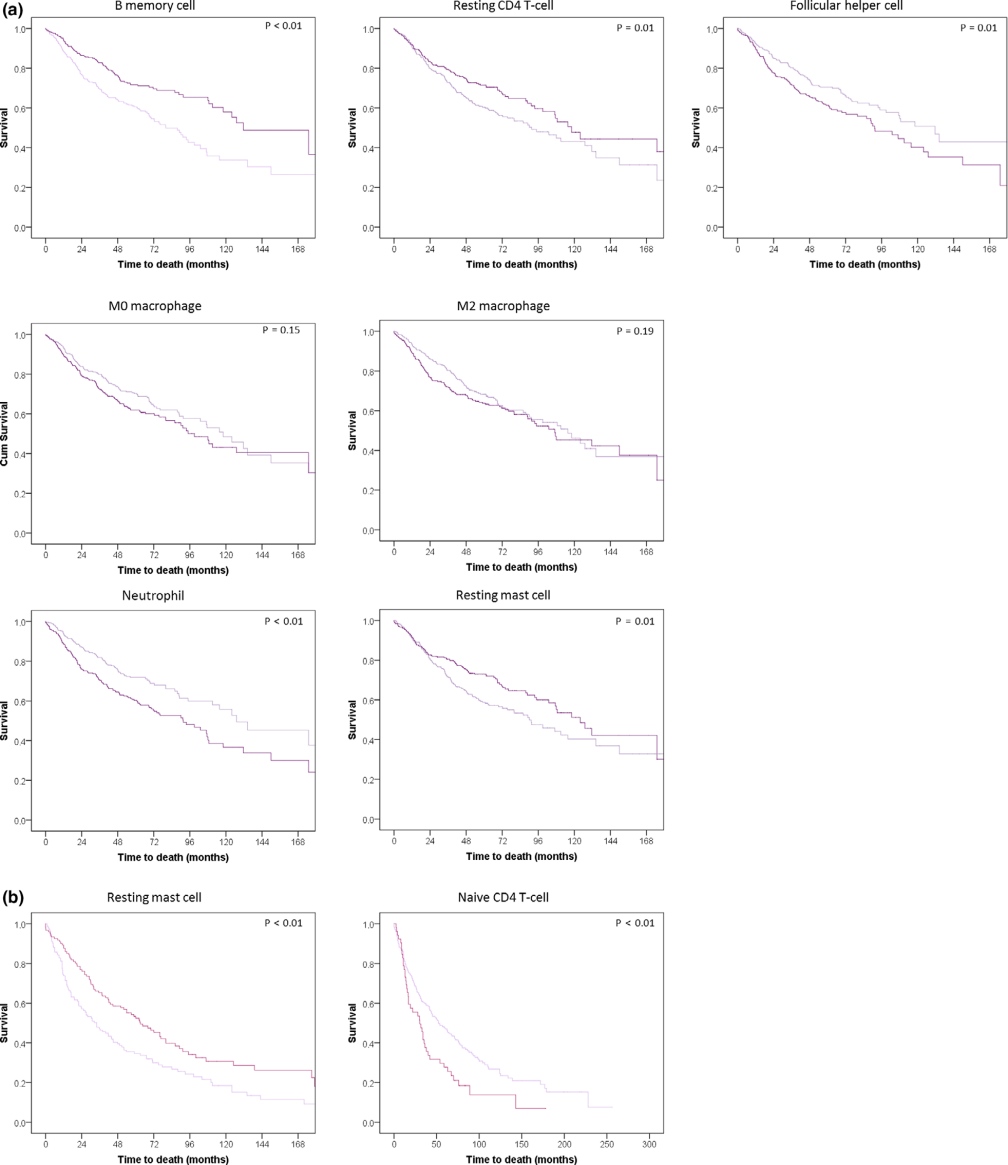
Survival curves of adenocarcinoma **(a)** (*N* = C587) and squamous cell carcinoma **(b)** (*N* = 254) NSCLC patients separated by the proportion of the cell fraction (above and below median). Survival curves of adenocarcinoma **(a)** and squamous cell carcinoma **(b)** patients split by their proportion (higher of lower than the median) of cell fractions. The faded line represents the patients with cell fractions below the median for both adenocarcinoma and squamous cell carcinoma. Depicted *P*‐values are those of the log‐rank test comparing the two groups split by the median proportion of the immune cell fraction.

For adenocarcinoma patients (*n* = 587), resting mast cell, memory B‐cell and resting CD4 T‐cell fractions were associated with better OS (HR = 0.96, *P = *0.01; HR = 0.97, *P = *0.01; HR = 0.98, *P = *0.04, respectively), while the neutrophil, follicular helper cell, M0 macrophage and M2 macrophage fractions were associated with a shorter OS (HR = 1.08, *P* < 0.01; HR = 1.07, *P = *0.01; HR = 1.02, *P = *0.01; HR = 1.02, *P = *0.03, respectively) (Figure 3, Supplementary table 7). For squamous cell carcinoma patients (*n* = 254), resting mast cells were associated with a better OS (HR = 0.94, *P = *0.01, Figures 3, 5, Supplementary table 8), while a higher percentage of regulatory T cells and naïve CD4 T cells were associated with a marginally poorer overall OS (HR = 1.12, *P = *0.06; HR = −1.1, *P = *0.06).

### Immune cell infiltrate by smoking status

Between smokers and non‐smokers, eleven cell fractions differed significantly (Figure 2). Smoking was associated with higher fractions of plasma cell (+2.9 percentage point), M0 macrophage (+2.8 percentage point), CD8 T‐cell (+1.4 percentage point) and follicular helper T cell (+0.9 percentage point) fractions, while the resting CD4 T‐cell (−4.1 percentage point), the resting mast cell (−3.1 percentage point), the memory B‐cell (−2.2 percentage point) and resting dendritic cell (−1.7 percentage point) fractions were lower compared to those in non‐smokers. The naïve B‐cell, active CD4 T‐cell and active mast cell fractions also differed significantly in their distribution.

### Survival impact of immune cell fractions by smoking status

**Figure 6:**
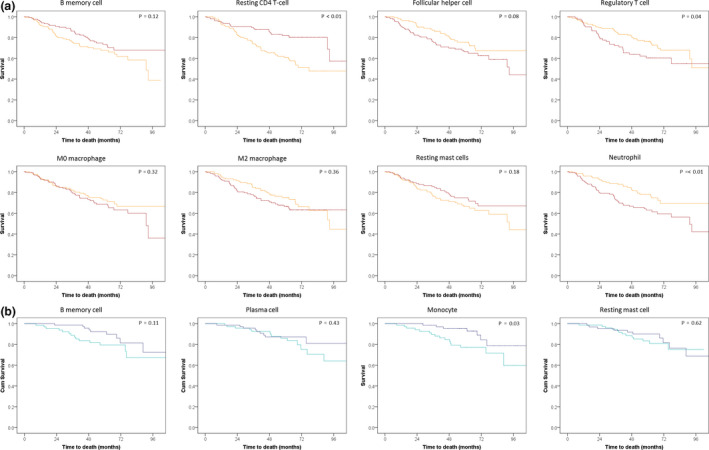
Survival curves of smoking **(a)** (*N* = 239) and non‐smoking **(b)** (*N* = 139) NSCLC patients separated by the proportion of the cell fraction (above and below median). Survival curves of smoking **(a)** and non‐smoking non‐small cell lung cancer **(b)** patients split by their proportion (higher of lower than the median) of cell fractions. For the smokers, the red line represents the above median proportion of the cell fraction and for the non‐smokers the dark blue line. For the non‐smokers, the naïve B‐cell fraction could not be split by its median and is therefore not shown. Depicted *P*‐values are those of the log‐rank test comparing the two groups split by the median proportion of the immune cell fraction.

For smokers (*n* = 572), we found that the resting mast cell, resting CD4 T‐cell and memory B‐cell fractions were associated with a better OS (HR = 0.9, *P = *0.01; HR = 0.92, *P* < 0.01; HR = 0.96, *P = *0.05, respectively), while the fractions of regulatory T cells, follicular helper T cells, neutrophils and M2 macrophages were associated with worse OS (HR = 1.27, *P = *0.01; HR = 1.12, *P = *0.02; HR = 1.09, *P = *0.04; HR = 1.04, *P = *0.02, respectively) (Figures 3, 6, Supplementary table 9). For non‐smokers (*n* = 148), the resting mast cell and memory B‐cell fractions were associated with better OS (HR = 0.9, *P = *0.07; HR = 0.92, *P = *0.04, respectively) while an increased proportion of plasma cells was associated with marginally worse OS (HR = 1.05, *P = *0.05) (Figure 3, Supplementary table 10).

### Clusters of immune cells

With different cluster analyses, no specific clusters of combination of immune cells or patients' groupings were identified that were associated with OS.

## Discussion

In this study, we have compared the immune microenvironment of the different histiotypes of lung cancer and of NSCLC patients smoking behaviour.

We observed that in NSCLC samples the immune cells consists mainly of plasma cells, macrophages, CD8 T cells, resting CD4 T cells and memory B cells. All were associated with (cancer‐related) overall survival, except for plasma cells. Between patients, large variations in immune fractions were observed. Others have shown that mononuclear phagocytes and T cells, especially regulatory T cells and non‐functional T cells, dominate in the early adenocarcinoma microenvironment.^25^, ^26^, ^27^, ^28^, ^29^, ^30^ Subtypes of NSCLC showed differences between immune fractions. Compared to squamous cell carcinoma, adenocarcinoma had higher fractions of memory B cells, resting mast cells and CD4 T cells. These cell fractions were associated with longer OS. Adenocarcinoma had lower percentages of M0 macrophages and neutrophils which were associated with worse survival. For squamous cell carcinoma, regulatory T cells and naïve CD4 T cells were associated with shorter survival, and both were lower compared to adenocarcinoma patients.

The subtype of NSCLC (adenocarcinoma vs squamous cell carcinoma) determines whether a specific cell fraction is associated with better or worse survival. Several cell fractions (neutrophil, memory B, naïve CD4 T) are negatively associated with survival for one subtype and positively for the other. These differences are in line with previous observations.^25^, ^26^, ^27^, ^28^, ^31^ Whether intratumoral exposure to neoantigens (squamous cell carcinoma patients are smokers and have a high tumor mutational burden) plays a role in the explanation of this phenomenon is not clear. Patients that (had) smoked showed higher fractions of cell types associated with immune regulatory functions like the M2 macrophages, regulatory T cells, neutrophils and follicular helper T cells. Smokers also showed higher fractions of plasma cells which were associated with shorter OS, concurring with the results of Alisoltani *et al*.^32^ The increased numbers of plasma cells could be due to the increased presence of neoantigens caused by the smoking behaviour. Plasma cells have an ambiguous role in cancer, as they have been both positively and negatively associated by different studies.^25^, ^32^, ^33^ Possibly smoking habits and histological subtype influence the survival outcomes, causing these different results. All of these cell fractions were associated with shorter survival in our study. Cell fractions associated with longer survival, specifically fractions of resting CD4 T cells, resting mast cells and memory B cells, were clearly lower in smokers compared to non‐smokers.

Clustering different proportions of immune cells does not show any groups of patients with similar immune infiltrate compositions. This result may be because there are no fixed cohorts of infiltration types or because the population is too heterogeneous.

Increased proportions of regulatory T cells are associated with poorer survival in smokers and squamous cell carcinoma patients. The cytotoxic activity of immune cells is negatively influenced by regulatory T cells and can occur without the actual presence of regulatory T cells in the tumor biopsy.^15^, ^34^, ^35^ The presence of regulatory T cells is an early event in the development of NSCLC.^36^ Together with neutrophils, they protect tumor cells against immune‐modulating effects.^37^ The infiltration of both neutrophils and regulatory T cells is induced by smoking. Smoking itself is also associated with both an increased frequency of infections and tissue inflammation.^36^, ^38^, ^39^, ^40^ That means that the infiltrate in smokers is composed of immune‐related cells that are triggered by various stimuli. This adds a complexity that makes the microenvironment in NSCLC tissue difficult to decipher the role of each immune cell in the tumor. In non‐smokers, the regulatory T‐cell population (and neutrophils) are less often present in the immune infiltrate, confirming that smoking is a confounding factor. Overall, smoking seems to induce an immune cell infiltrate that is less effective in suppressing tumor activity, because the differences in immune cell fractions in smokers compared to non‐smokers are associated with worse survival.

Cytotoxic CD8 T cells can be associated either with better or with worse survival, depending on the subtype of NSCLC.^34^, ^41^ Saito *et al*.^42^ found that the infiltration of CD8^+^ T cells throughout the tumor is associated with better survival, but their accumulation at one focal point is associated with the opposite, that is worse survival. This difference could have influenced our results, as it is likely that both types of CD8^+^ T‐cell invasion are present and cannot be distinguished in our study. Their opposing effects will diminish the association with survival. Furthermore, the contribution of exhausted or non‐functional CD8 T cells cannot be differentiated with the LM 22 algorithm. The different CD8 T‐cell functions are combined in our analysis and that may be an explanation that no overall survival differences are detected in our Cox regression model.

In our study, M2 macrophages (normally associated with wound healing and tissue repair) and neutrophils were significantly associated with worse survival. This indicates that M2 macrophages and neutrophils either have a tumor protective effect or possibly represent an ultimate attempt to fight the malignant cells that after all fails. Posttreatment studies have shown that M2 macrophages induce resistance to cisplatin therapy by means of activation of the JAK1/STAT1/NF‐κβ/Notch‐1 and ERK1/2/FRA‐1/slug signalling pathways, possibly explaining their negative association with survival.^43^, ^44^, ^45^ Their presence is believed to play an immune suppressive role, as it is associated with shorter survival and is negatively correlated to CD8^+^ T‐cell and T‐helper 1 cell infiltration.^46^ Neutrophils are associated with inactivated CD8 T cells, leading to worse outcomes.^47^, ^48^, ^49^, ^50^ However, their function remains ambiguous, as they have also been found to be capable of T‐cell activation. It is likely that specific subsets of neutrophils, TANs, influence survival in different ways. This remains a topic of interest for further studies.

Follicular helper T cells have been shown to strongly express PD‐1 and are important for the activation of effector cells in the lymph follicles.^51^ In NSCLC, studies found that follicular helper T cells present in tumor tissue were functionally impaired and associated with shorter disease‐free survival after resection.^52^, ^53^ The subsets of follicular helper T cells involved in NSCLC may be impaired in their normal function, causing less specific B‐cell differentiation and indirectly impaired humoral immune responses leading to tumor growth, explaining the worse survival association we found.

Resting mast cells are mostly known for their role in anaphylaxis by their release of histamine but also play a role in cancer immunity.^54^, ^55^ Histamine itself has been shown to stimulate tumor proliferation, while also suppressing the immune system.^54^, ^55^, ^56^ However, histamine might have a tumor‐suppressing effect when combined with IL‐6. Resting mast cells themselves are involved in tumorigenesis through the release of pro‐angiogenic factors and proteases involved in degeneration of the extracellular matrix.^56^, ^57^ However, mast cells also are involved in antitumor activity.^58^, ^59^, ^60^ When cancer progresses, the mast cells have limited capability to filtrate throughout the tumor, limiting their antitumor capabilities, which would explain while several studies in advanced cancers have reported an association between tumor growth and mast cells.^55^, ^56^

The limitations of our study are the measurement at a single pretreatment moment, with no data available at later time points, incomplete clinical datasets, working with cell fractions rather than absolute cell numbers (CIBERSORT has a high correlation to FACS outcomes [ρ = 0.97 in lung tissue]), a limited subset differentiation of cell types and functions, all inherent to our *in silico* approach. Additionally, the biopsy site (e.g. from the centre of the tumor or the edge) could have influenced the composition of the immune infiltrate, due to tumor heterogeneity. While most studies utilised similar guidelines to obtain biopsies and required a minimum number and percentage of tumor cells in the biopsy before they were processed for RNA, there remain considerable differences between patients. CIBERSORT resolved known mixture proportions over nearly the entire range of tumor content up to about 95% and noise up to about 70%. Since lung cancer often is composed of fewer than 50% infiltrating immune cells, the parameter range in which CIBERSORT outperformed other methods is highly relevant for bulk tumor analysis. By spike‐in experiments, it detects rare cells in bulk tissues down to 0.5% in mixtures containing up to 50% tumor content and down to 1% in mixtures over 50% tumor content.^61^ Studies with RNA‐seq and microarrays confirmed the robustness of CIBERSORT.^62^

In particular, we had limited data on smoking. Nevertheless, smoking has a major influence on the immune composition, but also on cell function. It is likely that cessation of smoking further modifies outcomes. Therefore, it is important for large prospective cohort studies to investigate the role of the immune system at several time points, focusing on cells suspected to be associated with survival and stratified for tumor subtype and smoking status. It could also be of interest to investigate differences in gender, as recently a study found survival differences depending on treatment.^63^

In conclusion, our study demonstrated that the immune cell infiltrate composition in NSCLC is associated with histological subtype and smoking. Variation between patient's tumors was large. Adenocarcinoma, as compared with squamous cell carcinoma, showed increased resting CD4 T cells and resting mast cells, both associated with longer survival, while having lower proportions of M2 macrophages and follicular helper T cells, associated with worse survival. Plasma cells in tumors had no impact on survival. For smokers, the resting CD4 T‐cell, memory B‐cell and resting mast cell fractions were all lower than those in non‐smokers and associated with longer survival, while neutrophils and regulatory T‐cell fraction were higher and associated with a shorter survival.

## Methods

### Data acquisition

Publically available raw microarray expression data were obtained by querying the Gene Expression Omnibus (GEO) (Supplementary table 1). The query was confined to samples hybridised to the Affymetrix HG‐U133 plus 2.0 (Geo accession number GPL570). After automatic querying, a second step was performed in which the identified samples were manually curated. Included samples had to be obtained by either biopsy or surgery so the whole tissue architecture was present. Sample exclusion occurred when sample description stated they were not derived from lung tissue, not from lung cancer; they were of foetal origin; cytological samples; cell lines; biopsies cultured; or subjected to treatment before or after removal. Clinical data such as gender, age, smoking status (current and past smoking versus non‐smoking), stage of disease, histology, treatment of the patients, Eastern Cooperative Oncology Group performance score, and overall survival data were collected when available. Missing data were requested from the corresponding authors.

### Sample processing and quality control

CEL files were obtained and checked for quality as reported previously.^64^ Non‐corrupted raw data CEL files were downloaded from GEO for the selected samples. To identify samples that have been uploaded to GEO multiple times, we generated a MD5 (message‐digest algorithm 5) hash for each individual CEL files. Before these MD5 hashes were generated, we converted all CEL files to the GCOS XDA binary file format (version 4), which was done using the Affymetrix Power Tools (version 1.15.2) apt‐cel‐ convert tool. A MD5 hash acts like a unique fingerprint for each individual file, and duplicate CEL files will have an identical MD5 hash. After removal of duplicate CEL files, pre‐processing and aggregation of CEL files were performed with RMAExpress (version 1.1.0) by applying the robust multi‐array average (RMA) algorithm, using the latest Affymetrix GeneChip Array CDF layout files REF. Principal component analysis (PCA) on the sample correlation matrix was used for quality control. The first principal component (PCqc) of such an expression microarray correlation matrix describes nearly always a constant pattern that dominates the data, explaining about 80–90% of the total variance, which is independent of the biological nature of the sample being profiled. The correlation of each individual microarray expression profile with this PCqc can be used to detect outliers, as arrays of lesser quality will have a lower correlation with the PCqc. We removed samples that had a correlation *R* < 0.8. All data were corrected using ComBat.

### Estimation of immune cell fractions in tumor microenvironment

The immune infiltrate composition was estimated using CIBERSORT, which uses gene expression profiles to characterise immune cell compositions of complex tissues by means of the LM22 signature matrix.^61^ The LM22 matrix contains 547 genes that distinguish 22 human haematopoietic cell phenotypes described in detail by Newman *et al*. (Supplementary table 2).^61^

### Statistical analysis

Differences in the distribution of immune cell fractions were compared with Mann–Whitney *U*‐tests. Test results with a *P* < 0.0022 (Bonferroni corrected) were considered significant. Associations with overall survival (OS) were assessed with multivariable Cox regression analyses. For the Cox regression variables, an event was defined as a death caused by lung cancer. Covariables were selected in a backwise model, with a stepwise exclusion of covariables with *P‐*values below 0.157 (based on Akaike information criterion). Covariables remaining in the model were age, gender, histological subtype, smoking status (current and past smoker, missing information, never smoker), and disease stage. Associations with OS have been reported in hazard ratios (HR). A HR > 1 indicates that a higher proportion of the immune cell is associated with worse OS, while a HR < 1 is associated with better outcome. As we used continuous variables, HR appears to be small. However, the provided HR is given for an increase of 1 percentage point of the immune cell fraction in question and stacks for every increment of 1 per cent. Both crude and adjusted values have been reported in the summary data (Supplementary tables 5–10), and associations with *P* ≤ 0.1 have been provided for NSCLC, subtype and smoking status.

Cox regression analyses were performed within a multivariate permutation testing framework for controlling the proportion of false discovery. For each subset analysis, we applied the multivariate permutation testing framework with 100 permutations and a false discovery rate (FDR) of 25%. An FDR of 25% indicates that the result is likely to be valid 3 out of 4 times.

To identify patient groups with comparable immune infiltrates, a k‐means clustering analysis was performed to identify those patients. All 22 immune cell fractions were incorporated. Schwarz's Bayesian criterion was used to assess the fit of the model. Subsequently, grouping variables were incorporated in the Cox regression analyses. All analyses were performed using IBM SPSS 23. In case of categorical variables, patients with missing data were grouped together (group = missing).

## Conflict of interest

The authors declare no conflict of interest.

## Supporting information

Supplementary figure 1
Supplementary tables 1–10

## Data Availability

The normalised data of the RNA sequencing, as well as the corresponding CIBERSORT outcomes and clinical data, are available on reasonable request from the Corresponding Author.
