# Supplementary material for: Immune microenvironment composition in non‐small cell lung cancer and its association with survival
Source: Clin Transl Immunology. 2020 Jun 12;9(6):e1142. doi: 10.1002/cti2.1142 (PMC7291326; doi:10.1002/cti2.1142)
Supplement: Supplementary file 1 — Supplementary figure 1 Supplementary tables 1–10 [file CTI2-9-e1142-s001.docx]

**Supplementary materials**:

Supplementary figure 1: Overall survival of mainly early NSCLC patients stratified by histological subtype


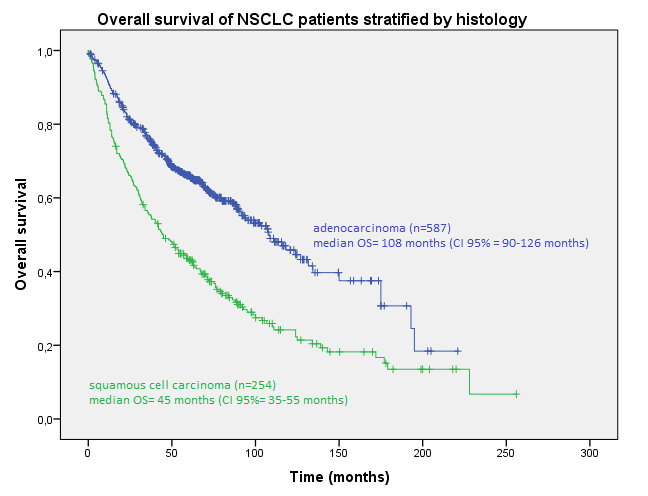


Patient numbers are for those with known histiotype and survival data.

| **Supplementary table 1: Search terms for GEO platform 571 and manual curation criteria** | |
| --- | --- |
| Search terms  (combined using OR) | Squamous  Adeno  NSCLC  SCLC  Lung  Non-small cell lung cancer  Long  Pulmonary |
| Manual selection criteria | Human lung tissue from normal and/or tumor biopsy  Exclusion of diseases that may influence the expression profile: (IPS/Sarcoidosis/COPD/HIV/Transplants)  Exclusion of all not-human tissue, amongst others cell lines  Exclusion of all tumor or normal tissue samples subjected to therapies |

| **Supplementary table 2: CIBERSORT defined cell type definitions** | | | |
| --- | --- | --- | --- |
|  | Markers used | Subdivisions | Identification |
| Naive B cells | CD3−, CD19+, CD20+, CD24−, CD38+ |  | |
| Memory B cells | CD3−, CD19+, CD20+, CD24+, CD38− |  |  |
| Plasma cells | CD19+, CD20+, CD138+ |  |  |
| CD8 T-cells | CD3+, CD8+ |  |  |
| Naive CD4 T-cells | CD3+, CD4+, CD45RA+, CD27+ |  |  |
| Memory CD4 T-cells | CD3+, CD4+, CD45RA− | Active | CD3+, CD4+, HLA-DR+ |
|  |  | Resting | Remaining memory cells |
| Follicular helper cells | CD3+, CXCR5^high^, ICOS^high^ |  | |
| Regulatory T-cells | CD3+, CD4+, FOXP3+ |  |  |
| Gamma delta T-cells | TCRgd+ |  |  |
| NK cells | CD16+, CD56+, CXCR3+ | Active | CD69+ |
|  |  | Resting | CD69- |
| Monocytes/Macrophages | CD14+ | Monocytes | Divided based on morphology and phagocytic capacity |
|  |  | M0 macrophages |  |
|  |  | M1 macrophages |  |
|  |  | M2 macrophages |  |
| Dendritic cells | CD14 isolation and stimulation by GM-CSF | Active | Lipopolysaccharides stimulation |
|  |  | Resting |  |
| Mast cells | CD 14 isolation and stem cell factor stimulation | Active | IgE receptor activation |
|  |  | Resting |  |
| Eosinophils | CD14+, CD15+, CD16- |  | |
| Neutrophils | CCR30-CD62+IgG |  |  |

| **Supplementary table 3: Studies with included samples** | |
| --- | --- |
| **Serie number GEO** | **Number of included samples** |
| GSE10245 | 58 |
| GSE10799 | 19 |
| GSE12667 | 69 |
| GSE16538 | 6 |
| GSE18842 | 45 |
| GSE19188 | 137 |
| GSE2109 | 98 |
| GSE21369/GSE21411 | 5 |
| GSE25251 | 2 |
| GSE27716/GSE27719 | 40 |
| GSE29013 | 2 |
| GSE29133 | 3 |
| GSE30219 | 206 |
| GSE31210 | 246 |
| GSE33532 | 76 |
| GSE3526 | 3 |
| GSE37745 | 172 |
| GSE40791 | 194 |
| GSE43580 | 148 |
| GSE50081 | 171 |
| GSE51024 | 39 |
| GSE7307 | 3 |
| Total | 1742 |

| Supplementary table 4: Available clinical data from 1430 NSCLC patients | | | | |
| --- | --- | --- | --- | --- |
| **Variable** | **Group** | **Samples with data** | **Values** | |
| **Age** | All NSCLC ((n=1430) | 1202 | Median (range) | 64 (30-93) |
|  | NSCLC with survival data (n=843) | 777 |  | 64 (30-88) |
|  | Adenocarcinoma (n=1022) | 842 |  | 64 |
|  | Squamous cell carcinoma (n=408) | 360 |  | 65 |
| **Gender** | All NSCLC (n=1430) | 1361 | Male/female | 843 / 518 |
|  | NSCLC with survival data (n=843) | 841 ( |  | 515 / 326 |
|  | Adenocarcinoma (n=1022) | 976 |  | 519/457 |
|  | Squamous cell carcinoma (n=408) | 385 |  | 324/61 |
| **Stage** | All NSCLC (n=1430) | 984 | I+II/III+IV | 922/62 |
|  | NSCLC with survival data (n=843) | 843 |  | 541/30 |
|  | Adenocarcinoma (n=1022) | 751 |  | 705/46 |
|  | Squamous cell carcinoma (n=408) | 233 |  | 217/16 |
| **Smoking** | All NSCLC (n=1430) | 752 | Smokers/  Non-smokers | 594/188 |
|  | NSCLC with survival data (n=843) | 378 |  | 239 / 139 |
|  | Adenocarcinoma (n=1022) | 604 |  | 170/434 |
|  | Squamous cell carcinoma (n=408) | 148 |  | 138/10 |

| **Supplementary table 5: Proportion of immune cells in non-small cell lung cancer tumors (n=1430)** | | | |
| --- | --- | --- | --- |
|  | **NSCLC** | | |
|  | Median | Minimum | Maximum |
| M2 macrophages | 17,52 | 0,00 | 53,75 |
| Resting mast cells | 3,55 | 0,00 | 38,10 |
| Resting CD4 T-cells | 5,39 | 0,00 | 31,64 |
| Plasma cells | 19,13 | 0,36 | 55,50 |
| CD8 T-cells | 6,85 | 0,00 | 34,06 |
| Monocytes | 0,00 | 0,00 | 23,34 |
| Neutrophils | 2,04 | 0,00 | 28,23 |
| Active NK cells | 3,65 | 0,00 | 17,00 |
| M0 macrophages | 5,21 | 0,00 | 45,09 |
| B memory cells | 5,36 | 0,00 | 32,01 |
| Active dendritic cells | 1,13 | 0,00 | 22,40 |
| M1 macrophages | 5,75 | 0,00 | 24,77 |
| Follicular helper cells | 2,97 | 0,00 | 13,65 |
| Resting dendritic cells | 2,52 | 0,00 | 31,65 |
| Resting NK cells | 0,00 | 0,00 | 16,99 |
| Naive B cells | 0,00 | 0,00 | 12,15 |
| Naive CD4 T-cells | 0,00 | 0,00 | 16,90 |
| Active CD 4 T-cells | 0,00 | 0,00 | 25,08 |
| Regulatory T-cells | 0,00 | 0,00 | 10,86 |
| Active mast cells | 0,00 | 0,00 | 30,76 |
| Eosinophils | 0,00 | 0,00 | 9,15 |
| Gamma delta T-cells | 0,00 | 0,00 | 21,60 |

| **Supplementary table 6: Correlation of immune cell fractions with survival in NSCLC patients (n=841), depicted as hazard ratios (HR)** | | | | |
| --- | --- | --- | --- | --- |
|  | Crude HR | Crude p value | Adjusted HR | Adjusted p value |
| Naive B cells | 1.02 | 0.55 | 0.97 | 0.28 |
| Memory B cells | 0.97 | 0.01 | 0.99 | 0.44 |
| Plasma cells | 1.00 | 0.88 | 0.99 | 0.17 |
| CD8 T-cells | 1.02 | 0.04 | 1.01 | 0.31 |
| Naive CD4 T-cells | 1.00 | 0.94 | 1.02 | 0.53 |
| Resting CD4 T-cells | 0.96 | 0.00 | 0.98 | 0.01 |
| Active CD 4 T-cells | 1.04 | 0.07 | 1.04 | 0.10 |
| Follicular helper cells | 1.08 | 0.00 | 1.05 | 0.01 |
| Regulatory T-cells | 1.11 | 0.01 | 1.07 | 0.09 |
| Gamma delta T-cells | 0.98 | 0.47 | 0.99 | 0.70 |
| Resting NK cells | 0.99 | 0.83 | 1.03 | 0.46 |
| Active NK cells | 0.98 | 0.33 | 0.99 | 0.66 |
| Monocytes | 0.94 | 0.06 | 0.99 | 0.59 |
| M0 macrophages | 1.03 | 0.05 | 1.02 | 0.02 |
| M1 macrophages | 1.03 | 0.05 | 1.01 | 0.57 |
| M2 macrophages | 1.02 | 0.03 | 1.01 | 0.07 |
| Resting dendritic cells | 0.99 | 0.52 | 0.99 | 0.48 |
| Active dendritic cells | 1.03 | 0.09 | 1.03 | 0.05 |
| Resting mast cells | 0.93 | 0.00 | 0.95 | 0.00 |
| Active mast cells | 1.02 | 0.25 | 1.01 | 0.56 |
| Eosinophils | 0.98 | 0.78 | 0.90 | 0.19 |
| Neutrophils | 1.05 | 0.02 | 1.03 | 0.08 |

| **Supplementary table 7: Correlation of immune cell fractions with survival in adenocarcinoma patients (n=587) depicted as hazard ratios (HR)** | | | | |
| --- | --- | --- | --- | --- |
|  | Crude HR | Crude p value | Adjusted HR | Adjusted p value |
| Naive B cells | 1.04 | 0.42 | 0.99 | 0.81 |
| Memory B cells | 0.96 | 0.00 | 0.97 | 0.01 |
| Plasma cells | 1.00 | 0.75 | 1.00 | 0.44 |
| CD8 T-cells | 1.01 | 0.53 | 1.01 | 0.37 |
| Naive CD4 T-cells | 0.91 | 0.16 | 0.92 | 0.18 |
| Resting CD4 T-cells | 0.97 | 0.01 | 0.98 | 0.04 |
| Active CD 4 T-cells | 1.03 | 0.22 | 1.04 | 0.14 |
| Follicular helper cells | 1.07 | 0.02 | 1.07 | 0.01 |
| Regulatory T-cells | 1.07 | 0.27 | 1.05 | 0.41 |
| Gamma delta T-cells | 1.01 | 0.88 | 1.01 | 0.69 |
| Resting NK cells | 0.95 | 0.52 | 1.00 | 0.97 |
| Active NK cells | 0.97 | 0.32 | 0.99 | 0.61 |
| Monocytes | 0.96 | 0.26 | 0.97 | 0.39 |
| M0 macrophages | 1.02 | 0.01 | 1.02 | 0.01 |
| M1 macrophages | 1.02 | 0.33 | 1.02 | 0.25 |
| M2 macrophages | 1.02 | 0.04 | 1.02 | 0.03 |
| Resting dendritic cells | 1.00 | 0.79 | 0.99 | 0.31 |
| Active dendritic cells | 1.04 | 0.16 | 1.04 | 0.10 |
| Resting mast cells | 0.94 | 0.00 | 0.96 | 0.01 |
| Active mast cells | 1.02 | 0.30 | 1.02 | 0.44 |
| Eosinophils | 1.05 | 0.72 | 0.99 | 0.95 |
| Neutrophils | 1.09 | 0.00 | 1.08 | 0.00 |

| **Supplementary table 8: Correlation of immune cell fractions with survival in squamous cell carcinoma patients (n=254), depicted as hazard ratios (HR)** | | | | |
| --- | --- | --- | --- | --- |
|  | Crude HR | Crude p value | Adjusted HR | Adjusted p value |
| Naive B cells | 0.95 | 0.27 | 0.95 | 0.24 |
| Memory B cells | 1.02 | 0.22 | 1.02 | 0.10 |
| Plasma cells | 0.99 | 0.29 | 0.99 | 0.30 |
| CD8 T-cells | 1.02 | 0.17 | 1.01 | 0.34 |
| Naive CD4 T-cells | 1.11 | 0.05 | 1.11 | 0.06 |
| Resting CD4 T-cells | 0.98 | 0.22 | 0.98 | 0.29 |
| Active CD 4 T-cells | 1.04 | 0.32 | 1.03 | 0.45 |
| Follicular helper cells | 1.03 | 0.36 | 1.02 | 0.59 |
| Regulatory T-cells | 1.16 | 0.01 | 1.12 | 0.04 |
| Gamma delta T-cells | 0.97 | 0.45 | 0.97 | 0.42 |
| Resting NK cells | 1.00 | 0.99 | 1.06 | 0.32 |
| Active NK cells | 1.02 | 0.51 | 1.01 | 0.81 |
| Monocytes | 1.01 | 0.85 | 1.05 | 0.47 |
| M0 macrophages | 0.99 | 0.38 | 1.00 | 0.90 |
| M1 macrophages | 1.00 | 0.86 | 0.99 | 0.64 |
| M2 macrophages | 1.01 | 0.32 | 1.01 | 0.36 |
| Resting dendritic cells | 1.02 | 0.37 | 1.01 | 0.62 |
| Active dendritic cells | 1.02 | 0.51 | 1.03 | 0.40 |
| Resting mast cells | 0.95 | 0.03 | 0.94 | 0.01 |
| Active mast cells | 0.99 | 0.61 | 0.99 | 0.96 |
| Eosinophils | 0.92 | 0.42 | 0.87 | 0.21 |
| Neutrophils | 0.99 | 0.70 | 0.99 | 0.61 |

| **Supplementary table 9: Correlation of immune cell fractions with survival in smoking NSCLC patients (n=239), depicted as the hazard ratios (HR)** | | | | |
| --- | --- | --- | --- | --- |
|  | Crude HR | Crude p value | Adjusted HR | Adjusted p value |
| Naive B cells | 0.97 | 0.66 | 0.96 | 0.61 |
| Memory B cells | 0.96 | 0.06 | 0.95 | 0.05 |
| Plasma cells | 1.00 | 0.79 | 1.00 | 0.72 |
| CD8 T-cells | 1.03 | 0.12 | 1.03 | 0.12 |
| Naive CD4 T-cells | 1.01 | 0.92 | 1.01 | 0.96 |
| Resting CD4 T-cells | 0.92 | 0.00 | 0.92 | 0.00 |
| Active CD 4 T-cells | 1.01 | 0.77 | 1.01 | 0.72 |
| Follicular helper cells | 1.12 | 0.02 | 1.12 | 0.02 |
| Regulatory T-cells | 1.27 | 0.01 | 1.27 | 0.01 |
| Gamma delta T-cells | 0.98 | 0.74 | 0.98 | 0.76 |
| Resting NK cells | 0.68 | 0.11 | 0.69 | 0.12 |
| Active NK cells | 0.99 | 0.85 | 0.99 | 0.81 |
| Monocytes | 0.90 | 0.21 | 0.91 | 0.24 |
| M0 macrophages | 1.02 | 0.10 | 1.02 | 0.13 |
| M1 macrophages | 1.03 | 0.23 | 1.04 | 0.14 |
| M2 macrophages | 1.04 | 0.03 | 1.04 | 0.02 |
| Resting dendritic cells | 0.98 | 0.50 | 0.98 | 0.43 |
| Active dendritic cells | 1.00 | 0.94 | 1.00 | 0.93 |
| Resting mast cells | 0.92 | 0.01 | 0.92 | 0.01 |
| Active mast cells | 0.99 | 0.79 | 0.99 | 0.81 |
| Eosinophils | 0.84 | 0.57 | 0.86 | 0.65 |
| Neutrophils | 1.10 | 0.02 | 1.09 | 0.04 |

| **Supplementary table 10: Correlation of immune cell fractions with survival in non-smoking NSCLC patients (n=139)** | | | | |
| --- | --- | --- | --- | --- |
|  | crude HR | crude p value | adjuster HR | adjusted p value |
| Naive B cells | 1.24 | 0.01 | 1.15 | 0.09 |
| Memory B cells | 0.93 | 0.04 | 0.92 | 0.04 |
| Plasma cells | 1.02 | 0.28 | 1.05 | 0.05 |
| CD8 T-cells | 0.94 | 0.29 | 0.96 | 0.50 |
| Naive CD4 T-cells | 0.93 | 0.65 | 0.89 | 0.46 |
| Resting CD4 T-cells | 1.02 | 0.60 | 0.99 | 0.78 |
| Active CD 4 T-cells | 1.14 | 0.41 | 1.21 | 0.24 |
| Follicular helper cells | 0.96 | 0.70 | 1.04 | 0.75 |
| Regulatory T-cells | 0.69 | 0.23 | 0.83 | 0.52 |
| Gamma delta T-cells | 1.00 | 0.93 | 1.03 | 0.75 |
| Resting NK cells | 1.02 | 0.95 | 1.16 | 0.60 |
| Active NK cells | 0.93 | 0.38 | 0.94 | 0.48 |
| Monocytes | 0.89 | 0.33 | 0.80 | 0.09 |
| M0 macrophages | 1.01 | 0.58 | 1.03 | 0.22 |
| M1 macrophages | 1.04 | 0.39 | 1.02 | 0.72 |
| M2 macrophages | 1.00 | 0.84 | 0.98 | 0.52 |
| Resting dendritic cells | 1.04 | 0.31 | 1.00 | 0.95 |
| Active dendritic cells | 1.02 | 0.86 | 1.06 | 0.51 |
| Resting mast cells | 0.94 | 0.18 | 0.90 | 0.07 |
| Active mast cells | 1.02 | 0.80 | 1.07 | 0.32 |
| Eosinophils | 1.41 | 0.24 | 1.28 | 0.44 |
| Neutrophils | 1.07 | 0.41 | 1.1 | 0.27 |
